# Minimal change nephrotic syndrome in an 82 year old patient following a tetanus-diphteria-poliomyelitis-vaccination

**DOI:** 10.1186/1471-2369-10-21

**Published:** 2009-08-05

**Authors:** Christian Clajus, Janine Spiegel, Verena Bröcker, Christos Chatzikyrkou, Jan T Kielstein

**Affiliations:** 1 Department of Nephrology and Hypertension, Medical School Hannover, Hannover, Germany; 2 Institute of Pathology, Medical School Hannover, Hannover, Germany

## Abstract

**Background:**

The most common cause of idiopathic nephrotic syndrome in children and younger adults is the minimal change nephrotic syndrome (MCNS). In the elderly MCNS is relatively uncommon. Over the last decade some reports suggest a rare but possible association with the administration of various vaccines.

**Case presentation:**

A 82-year old Caucasian female presented with pronounced nephrotic syndrome (proteinuria of 7.1 g/d, hypoproteinemia of 47 g/l). About six weeks prior to admission, she had received a combination vaccination for tetanus, diphtheria and poliomyelitis as a booster-vaccination from her general practitioner. The renal biopsy revealed typical minimal change lesions. She responded well to the initiated steroid treatment. As through physical examination as well as extensive laboratory and imaging studies did neither find any evidence for malignancies nor infections we suggest that the minimal change nephrotic syndrome in this patient might be related to the activation of the immune system triggered by the vaccination.

**Conclusion:**

Our case as well as previous anecdotal reports suggests that vaccination and the resulting stimulations of the immune system might cause MCNS and other severe immune-reactions. Increased awareness in that regard might help to expand the database of those cases.

## Background

The most common cause of idiopathic nephrotic syndrome in children and younger adults is the minimal change nephrotic syndrome (MCNS). In the elderly MCNS is relatively uncommon. If present it can be associated with several conditions as malignancies [1], viral infections, allergies [2] and various drugs. Over the last ten years four anecdotal reports linking MCNS to vaccinations against hepatitis [3-5] pneumococcus [6] and influenca [7] have been published. Here we report the first case of MCNS following a combined tetanus-diphteria-poliomyelitis-vaccination and also summarize all previous published reports on MCNS after vaccination.

## Case presentation

An 82 year old Caucasian female was admitted to our hospital in April 2008 with pitting edema of her legs arms and back. Her past medical history was significant for an appendectomy at the age of 29 years, a hyster- and ovariectomy at the age of 50 years, as well as status post varicose stripping, bilateral knee arthrosis, hypercholesterolemia and a reported diphtheria infection as a child. No history of allergic diseases or malignoma was present. Her regular medication consisted of low dose acetylsalicylic acid (100 mg/d), simvastatine (5 mg/d) and occasionally diclofenac (about 3 times a year, last time four months prior to admission). The outpatient nephrologist started medical therapy with spironolactone (25 mg) and furosemide (up to 400 mg/d) three days prior to admission. Laboratory data from her routine outpatient visits showed no proteinuria as well as normal renal function until January 2007.

On January 17^th ^2008, the patient received a combined intramuscular vaccination for tetanus, diphtheria and poliomyelitis (REVAXIS, Sanofi Pasteur MSD GmbH, Leimen, Germany), as her last vaccination for tetanus had been performed in April 1998. The vaccination was composed of cleaned tetanus toxoid, cleaned diphtheria toxoid, inactive poliomyelitis virus Typ 1–3, aluminiumhydroxide, 2-Phenoxyethanol, formaldehyde, medium 199 (mixture of aminoacids, minerals, vitamins, polysorbat 80) and trace amounts of neomycin, streptomycin and polymyxin B. No local vaccination reaction occurred after injection. At the end of February slight leg edema occurred. The general practitioner performed echocardiography for suspected heart failure. As the echocardiography revealed only mild aortic-valve-insufficiency and mild mitral valve-insufficiancy, with an intact overall contractility this diagnosis was dismissed. Meanwhile the edema worsened constantly, impressively indicated by an increase in bodyweight from 72 kg to 84 kg in one month. Moreover, the previous normotensive patient became hypertensive with blood pressure of 186/101 mmHg. Urinary dip-stick analysis now showed massive (+++) proteinuria on which the patient was transferred to a nephrologist. The 24 hours collected urine showed massive unselective proteinuria (12 g/day), therapy with spironolactone and furosemide failed to alleviate the edematous state. The urine sediment revealed multiple erythrocyte cylinders on which the patient was transferred to our tertiary care hospital for further nephrological evaluation and treatment. Upon admission physical examination was significant for general edema and multiple localised makulopapulous exanthema of the feet and legs. Vital parameters were unremarkable. The chest radiograph showed no signs of pleural effusions or malignancies. Laboratory tests revealed unselective nephrotic proteinuria (7.13 g/d), hypoproteinemia (47 g/l) and hypercholesterolemia (605 mg/dl). Serum creatinine was 74 μmol/l. The 24 hour collected urine revealed a reduced creatinine-clearance of 46 ml/min. Urinary sediment showed mild microhaematuria (5–10 erythrocytes/μl) and a few granulated and hyaline cylinders but no signs of an active sediment. Analysis for antinuclear antibodies, ANCA, antibodies against double-strain-DNS, anti-GBM-antibodies and Hepatitis B and C were negative or within normal limits. The kidneys were of normal size but showed bilateral mild enhancement in the abdominal ultrasound. Further diagnostic workup showed no evidence of malignancies.

**Figure 1:**
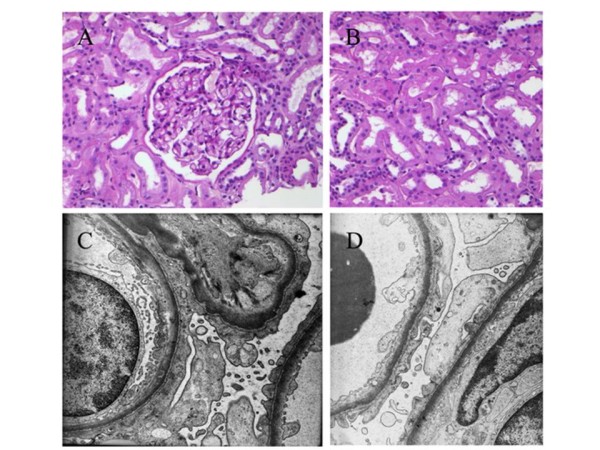
**Light-microscopy of a representative Glomerulum and the tubulus-interstitium (A, B) and ultrastructure of a glomerulum with missing podocytes and thin basements without deposits (C, D)**.

Renal biopsy was performed and showed typical minimal change lesion without evidence of focal and segmental glomerulosclerosis (Figure 1). Three of the 22 glomerula showed globalsclerosis, minimal tubulo-atrophie and interstitial fibrosis (< 5% of the cortex). Further immunological staining excluded mesangial and glomerular IgM, IgA, IgG, C3 or fibrin/fibrinogen deposits.

**Figure 2:**
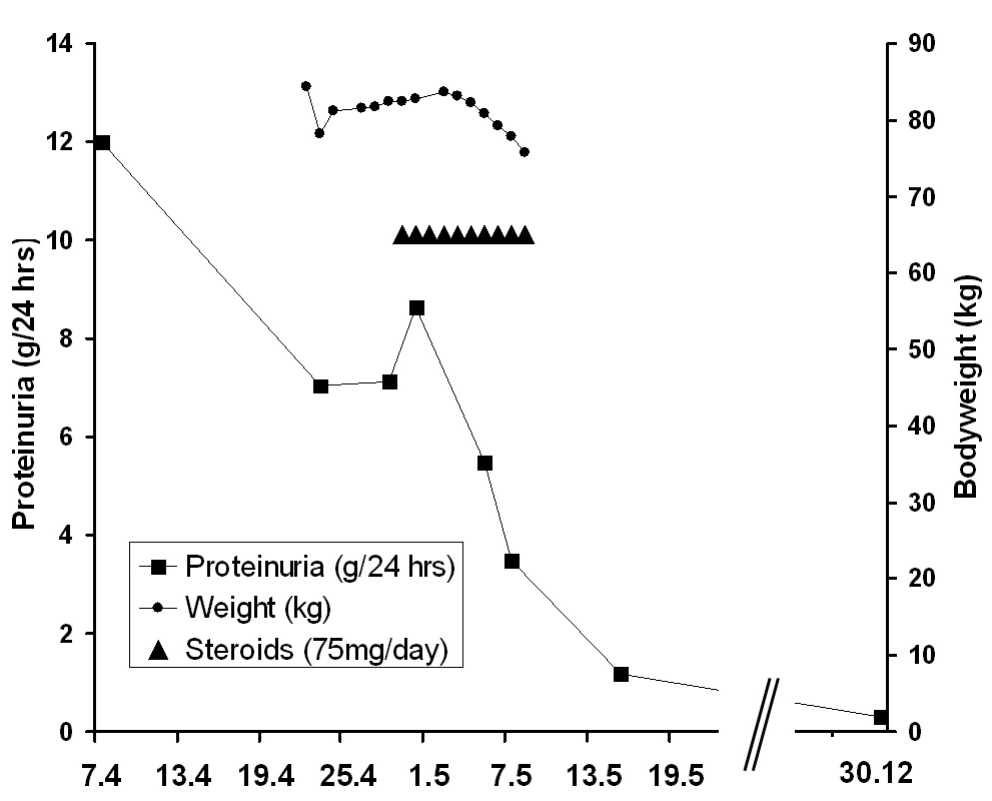
**Clinical course of the patient**.

After minimal change diagnosis was assured by biopsy, therapy with steroids (1 mg/kg body weight/day) and an ACE-inhibitor (ramipril 5 mg/d) was initiated. After ten days of therapy with steroids (75 mg/d) with concomitant oral phenprocourmon the nephrotic range proteinuria reduced to 1.17 g/24 h (Figure 2). On the last follow-up in the end of April 2009 serum-creatinine fell to 62 μmol/l, the blood pressure normalized and proteinuria was not detectable in a 24 h urine collection.

## Methods

For this paper we searched PubMed for relevant articles using the following medical subject headings: "minimal change nephropathy," "vaccination," through Feburary 1, 2009. We supplemented the search by scanning references of selected articles identified by this search.

## Conclusion

Minimal change nephrotic syndrome (MCNS) is a major cause of nephrotic syndrome in both children and younger adults. Although the exact underlying cause of MCNS is not fully understood a burgeoning body of evidence suggests that systemic T cell dysfunction results in the production of a glomerular permeability factor. This circulating factor directly induces foot process fusion resulting in severe alteration of the glomerular filter system and resulting in marked proteinuria. The clinical and histopathological findings in our patient as well as the course of the disease were typical for MCNS. This syndrome is rarely seen in elderly patients (3/million/year) [8]. The fact that our patient did not exhibit any other condition known to be associated with MCNS at the time of presentation nor on follow up, suggests that the vaccination might have caused MCNS in our patient.

To date six reports of MCNS after vaccination had been published, including our report (Table 1). The first published evidence for a nephrotic syndrome following vaccination is given in an article from Chamberlain et. al in 1966 [9]. A woman suffered a nephrotic syndrome 10 days after smallpox vaccination. Biopsy and later necropsy was performed showing slight basement thickening making MCGN an unlikely diagnosis. In all presented cases the patients suffered a nephrotic syndrome shortly after vaccination of different sera (Tetanus-diphteria-poliomyelitis; Pneumococcus; Influenza; Hepatitis B). In 4 cases diagnosis was assured by biopsy, except one patient all others were treated with steroids. In three of the published cases follow-up phase was negative concerning proteinuria. Our patient reported the occasional use of diclofenac. Non-steroidal-anti-rheumatic-drugs (NSAID's) are well known to cause MCGN as Galesic et al have recently published [10]. In our case this cause is unlikely because the patient denied the use of any NSAID four month prior to the admission.

**Table 1:** Summary of MCNS following vaccination in literature

Vaccination against	Tetanus-diphteria-poliomyelitis-vaccination	Pneumococcus [6]	Influenza [7]	Hepatitis B [3]	Hepatitis B [4]	Hepatitis B [5]
Age [years]	82	67	65	3	40	4
Gender	female	female	female	male	female	male
Baseline creatinine	76 μmol/l	no data	normal	44 μmol/l	normal	no data
Peak creatinine	138 μmol/l	274 μmol/l	158 μmol/l	no data	no data	no data
Baseline proteinuria	negative in dip-stick	past history unremarkable	no data	past history unremarkable	past history unremarkable	past history unremarkable
Peak proteinuria	12 g/day	10.4 g/day	13.2 g/day	24.8 g/day	8 g/day	1.25 g/day
Vaccination to onset of symptoms	4 weeks	4 months	4 days	17 days	after 2^nd ^inoculation	8 days
Biopsy	typical minimal change lesion (MCL)	MCL and mild interstitial nephritis	typical minimal change lesion (MCL)	not indicated	minimal change nephropathy	not indicated
Treatment	Steroids 1 mg/kg bw ACE-inhibitor	750 mg Steroids for 3 days; followed by 40 mg/day	None specific	Steroids 2 mg/kg bw	Steroids (12 mg every other day)	Steroids 2 mg/kg bw
Renal function/follow up	80 μmol/l 6 months after diagnosis	Urinary protein neg. after one year; 15 mg Steroids/day	Clearacnce 95 ml/min after one year	no data	no data	Complete remission

On admission in our hospital the patient complained of multiples makulopapulous exanthema on her legs which shortly occurred after the ambulatory start of the diuretic therapy with furosemide. As furosemide is known to cause skin exanthema [11] and the lesions disappeared after discontinuation of the loop-diuretic, we attribute the skin lesions to the furosemide administration as an allergic drug exanthema and not as an erythema exsudativum multiforme as a sign for an infection. The allergic origin is supported by the fact that was a lag period between the clinical signs of the nephritic syndrome and a temporal association between the begin of the furosemid therapy and the begin of the exanthema.

In our patient the time of onset of the nephrotic syndrome after the vaccination correlates with the reported time at which seroprotective antibody-levels are confirmed after vaccination with REVAXIS (28 days after injection) [12,13]. The time until the onset of symptoms varies in all described cases cases from days to months, which could be explained by the different time it took for the respective vaccine to trigger an immune response. Nephrotic syndrome occurred after the second administration of the vaccine which suggests that the last administration has boosted a pre-existing immune response from the first vaccination. Similar findings are described in a case from Floege et al. [19].

Another serious reaction after vaccination on renal function is described in several cases. Santoro reported a Lupus nephritis as an uncommon complication following a hepatitis B vaccination [14]. In another case from Poland, a 17-year-old girl suffered a necrotizing glomerulonephritis after a vaccination against influenza, which underlines the hypothesis of the activation of the immune-system after vaccination [15].

Should we than stop vaccinations, at least for children with nephrotic syndrome? In a study assessing the relapse rate of 54 children with known steroidsensitive nephrotic syndrome showed no evidence that meningococcal C conjugate vaccine (MCCV) triggered relapses of nephrotic syndrome [16]. This is in contrast to findings by Abeyagunawardena and colleagues who advised to carefully consider the vaccination of children with steroid-sensitive nephrotic syndrome. In their cohort of 106 children with nephrotic syndrome showed a significantly higher relapse rate after MCCV-vaccination with a relative incidence of 1.52 (95% CI 1·10–2·11) comparing 12 months pre- and post-vaccination [17]. This is in line with a another study showing a nephrotic syndrome relapse rate of 9% after vaccination against viral hepatitis B in children [18]. In summary, various vaccinations are associated with a higher relapse of steroid-sensitive nephrotic syndrome, yet the benefit for the preventive medicine prevails the risks of triggering a relapse these patients. The currently available data do in our view not allow giving any firm recommendation concerning the use vaccines patients with nephrotic syndrome. In the general population the benefit of various vaccines has been clearly documented as an effective way of primary prevention of diseases. Extremely rare side effects of vaccinations, like MCNS, can by no means outweigh the benefits of vaccinations.

It is beyond the scope of a case report to elucidate the possible pathophysiology between the immune response after vaccination in MCNS. Still one could hypothesize that under certain preconditions, the immune response after vaccination influences the cytosceleton of podocytes, leading to proteinuria. Indeed, recent publications showed that the podocyte is not only heavily equipped with cytokine receptors but is also a target of immunosuppressive drugs like cyclosporin. Hence, podocyte integrity could, under certain preconditions, be altered in the context of a stimulated immune system after vaccination.

## Competing interests

The authors declare that they have no competing interests.

## Authors' contributions

CC, JS, CC and JTK were the treating physicians of the patient reported. VB performed the evaluation of the renal biopsy. All of the authors have participated in the discussion and in writing of the submitted manuscript.

## Pre-publication history

The pre-publication history for this paper can be accessed here:

http://www.biomedcentral.com/1471-2369/10/21/prepub

## References

[B1] Eagen JW Glomerulopathies of neoplasia Kidney Int 1977 11 297 303 10.1038/ki.1977.47 197291

[B2] Sandberg DH Bernstein CW McIntosh RM Carr R Strauss J Severe steroid-responsive nephrosis associated with hypersensitivity Lancet 1977 1 388 391 10.1016/S0140-6736(77)92603-4 65510

[B3] Ozdemir S Bakkaloglu A Oran O Nephrotic syndrome associated with recombinant hepatitis B vaccination: a causal relationship or just a mere association? Nephrol Dial Transplant 1998 13 1888 1889 10.1093/ndt/13.7.1889b 9681759

[B4] Macario F Freitas L Correia J Campos M Marques A Nephrotic syndrome after recombinant hepatitis B vaccine Clin Nephrol 1995 43 349 7634556

[B5] Islek I Cengiz K Cakir M Kucukoduk S Nephrotic syndrome following hepatitis B vaccination Pediatr Nephrol 2000 14 89 90 10654341

[B6] Kikuchi Y Imakiire T Hyodo T Higashi K Henmi N Suzuki S Miura S Minimal change nephrotic syndrome, lymphadenopathy and hyperimmunoglobulinemia after immunization with a pneumococcal vaccine Clin Nephrol 2002 58 68 72 12141410 10.5414/cnp58068

[B7] Kielstein JT Termuhlen L Sohn J Kliem V Minimal change nephrotic syndrome in a 65-year-old patient following influenza vaccination Clin Nephrol 2000 54 246 248 11020024

[B8] Sharpstone P Ogg CS Cameron Nephrotic syndrome due to primary renal disease in adults: I. Survey of incidence in South-east England Br Med J 1969 2 533 535 10.1136/bmj.2.5656.533 5769886 PMC1983477

[B9] Chamberlain MJ Pringle A Wrong OM Oliguric renal failure in the nephrotic syndrome Q J Med 1966 35 215 235 5912056

[B10] Galesic K Ljubanovic D Bulimbasic S Racic I Minimal change disease and acute tubular necrosis caused by diclofenac Nephrology (Carlton) 2008 13 87 88 18199110 10.1111/j.1440-1797.2007.00863.x

[B11] Thestrup-Pedersen K Adverse reactions in the skin from anti-hypertensive drugs Dan Med Bull 1987 34 Suppl 1 3 5 2893692

[B12] Laroche P Barrand M Wood SC Van HK Lang J Harzer E Hessel L The immunogenicity and safety of a new combined diphtheria, tetanus and poliomyelitis booster vaccine (Td-eIPV) Infection 1999 27 49 56 10.1007/BF02565175 10206791

[B13] Stojanov S Liese JG Bendjenana H Harzer E Barrand M Jow S Dupuy M Belohradsky BH Immunogenicity and safety of a trivalent tetanus, low dose diphtheria, inactivated poliomyelitis booster compared with a standard tetanus, low dose diphtheria booster at six to nine years of age. Munich Vaccine Study Group Pediatr Infect Dis J 2000 19 516 521 10.1097/00006454-200006000-00005 10877165

[B14] Santoro D Stella M Montalto G Castellino S Lupus nephritis after hepatitis B vaccination: an uncommon complication Clin Nephrol 2007 67 61 63 17269603 10.5414/cnp67061

[B15] Hyla-Klekot L Kucharska G Cieslak W [Necrotizing glomerulonephritis in decursu vasculitis after vaccination against influenza] Pol Merkur Lekarski 2005 19 75 77 16194032

[B16] Taylor B Andrews N Stowe J Hamidi-Manesh L Miller E No increased risk of relapse after meningococcal C conjugate vaccine in nephrotic syndrome Arch Dis Child 2007 92 887 889 10.1136/adc.2006.105247 17468130 PMC2083230

[B17] Abeyagunawardena AS Goldblatt D Andrews N Trompeter RS Risk of relapse after meningococcal C conjugate vaccine in nephrotic syndrome Lancet 2003 362 449 450 10.1016/S0140-6736(03)14072-X 12927434

[B18] Szajner-Milart I Zajaczkowska M Zinkiewicz Z Borzecka H Majewski M Efficacy of vaccination against viral hepatitis type B in children with the nephrotic syndrome Lancet 2003 58 402 408 15315023

[B19] Floege J Lonnemann G Stichtenoth DO Koch KM Brunkhorst R Minimal change nephrotic syndrome in a 74-year-old patient following parenteral administration of sheep cells Nephrol Dial Transplant 1998 13 1484 1488 9481738 10.1093/ndt/13.6.1484

